# Exacerbation of hepatitis C induced subclinical hypoadrenalism by Interferon-alpha2beta: A case report

**DOI:** 10.1186/1757-1626-1-157

**Published:** 2008-09-18

**Authors:** Huy A Tran, Shuzhen Song, Robert J Lojewski, Glenn E Reeves

**Affiliations:** 1 Department of Clinical Chemistry, Hunter Area Pathology Service, Locked Bag Number 1, Hunter Mail Region Centre, Newcastle, New South Wales 2310, Australia; 2 Department of Immunopathology, Hunter Area Pathology Service, Locked Bag Number 1, Hunter Mail Region Centre, Newcastle, New South Wales 2310, Australia

## Abstract

Adrenal disease is an uncommon manifestation of hepatitis C infection and its related treatment regimen. This is a case of subclinical hypoadrenalism, probably induced by hepatitis C infection and further exacerbated by interferon-α2β and Ribavirin therapy. The adrenal deterioration during the treatment course was observed closely with 24-hour salivary profiles and 250 μg adrenocorticotropin stimulation tests using parallel serum and salivary cortisol concentrations. A number of possible pathogenic mechanisms are discussed, and the controversy over its management is emphasized.

## Case presentation

A 55 year old post menopausal Caucasian female presented with vitiligo on her face, arms and legs. Her past medical history included only mild asthma requiring only intermittent bronchodilators without glucocorticoids. There was no other significant personal or family medical history. Clinical examination showed a well woman, weight of 69.1 kg, height 1.67 metre (body mass index ~25). Her blood pressure was 120/75 lying and 120/70 sitting with a regular pulse of 78 beats per minute. There were 3 vitiligo patches each measuring approximately 3 × 5 cm on her forehead, anterior abdomen and left cubital fossa. No goitre or liver enlargement was detected. Biochemical investigations are as follow: sodium 130 mmol/L (reference range (RR), 136–146), potassium 5.4 mmol/L (RR, 3.5–5.5), chloride 99 mmol/L (RR, 98–108), bicarbonate 21 mmol/L (RR, 24–30), urea 9.1 mmol/L (3.0–7.0), creatinine 90 umol/L (RR, 40–90), bilirubin 18 μmol/L (6–10), alanine aminotransferase 105 IU/L (RR, < 50), aspartate aminotransferase 56 IU/L (RR, < 45), alkaline phosphatase 136 IU/L (RR, < 110), γ-glutamyl transferase 40 IU/L (< 60), albumin 33 g/L (RR, 35–45), plasma aldosterone 172 pmol/L (RR, 80–1040), plasma renin activity > 35.7 ng/mL/hr (RR, 1.2–2.8). In essence, they showed mild hyperkalaemic metabolic acidosis and hepatocellular dysfunction. Because the latter persisted, chronic hepatitis C was confirmed with positive serology of genotype 1. The liver biopsy showed changes consistent with chronic persistent hepatitis, the inflammatory and fibrotic changes were both graded 1 according to the scoring method [1]. Together with additional biochemical and immunological studies, other causes of persistent abnormal liver function tests were excluded. In view of her vitiligo, hepatitis C and biochemical disturbance, the Adrenocorticotropin (ACTH) stimulation test (AST), also known as the Cosyntropin or Short Synacthen test, was performed which revealed the presence of subclinical adrenal insufficiency (AI), additional file 1. Further investigations for the causes of AI revealed positive adrenal cell antibody (ACA) serology. Computerised tomographic scan showed small adrenal glands bilaterally with normal anatomy and appearance.

The patient underwent routine combination treatment of Interferon-α2β (IFN) and Ribavirin (RBV) for 48 weeks for her genotype 1 HCV infection. The patient's subclinical adrenal disease was also followed closely using salivary as well as serum cortisol levels to assess the hypothalamo-pituitary-adrenal axis (HPA) every 12 weeks, starting at baseline, during treatment, 6 and 12 month follow-ups. The results suggested a progressive decline of her adrenal function during the treatment phase. Glucocorticoid replacement therapy was seriously considered but with apprehension due to possible exacerbation of the hepatitis. The risks were discussed in detail with the patient and her family and it was decided to continue with anti-viral therapy but without glucocorticoid supplement. The patient and her husband were counselled comprehensively regarding the emergency management of Addisonian crisis, provided with a carrying note and required to wear an alert bracelet. Other immediate family members were also involved in her management plan, with her consent. Her management plan was also forwarded to the local hospital Emergency Department and Hepatitis C Service. Fortunately, there was no crisis other than the common side effects of the treatment regimen. Her pattern of steroid profile and ACA returned to its pre-existing state and remained unchanged at 6 and 12 month follow-up after the cessation of therapy. The patient has remained well since but her subclinical AI persists.

## Discussion

Hepatitis C is well-documented to be associated with many auto-immune endocrinopathies, especially thyroid but also including the hypothalamus, pituitary, renal organs [2,3]. This is the first case to explore the challenge of subclinical AI and the influence of interferon treatment upon it. In the presence of positive ACA, it was very probable that AI is the result of HCV infection and its immunomodulating effect on adrenal tissue, as most alternative causes are not apparent.

It was interesting that patient was found to have subclinical AI in which the diagnosis was confirmed by the failure to stimulate an adequate cortisol rise after 60 minutes of Cosyntropin stimulation even though the baseline levels were satisfactory throughout. In healthy subjects, there must be an absolute increase of 300 nmol/L or more at the 60 minute sample [4]. Another definition of non-responders has been suggested to be less than a 20% increase compared with baseline value [5], directly relevant to this patient. Additionally, the elevated ACTH and renin levels and reduced aldosterone/renin ratio conclusively indicated adrenal cortical failure. Various stages of progressive adrenal failure have been described but this case clearly does not fit into any of the prescribed categories [6]. According to this proposal, our patient belongs in the *'Symptomatic Under Stress' *category in which she satisfied all criteria except that her baseline cortisol was normal rather less than 83 nmol/L.

During IFN and RBV therapy, there was a steady and gradual decline in baseline salivary and serum cortisol levels prior to ASTs. Serum ACTH levels increased slightly throughout the treatment course and returned to pre-treatment level after. The failure of cortisol levels to rise at 60 minutes is consistent in both saliva and serum throughout the 48 weeks of treatment. There was also a significant decrease in cortisol levels during therapy (additional file 1). At 12 months following the end of IFN therapy, the baseline cortisol levels returned to their pre-treatment levels.

The longitudinal 24-hour salivary cortisol profile throughout the course of treatment is rewarding and revealing. The normal physiological diurnal pattern has been lost with the deletion of the second late-afternoon cortisol peak. Although the first morning peak was retained, it was significantly attenuated to a mean of 6.2 nmol/L followed by a gradual decline to an undetectable nadir at midnight. The effect and influence of IFN on the 24-hour salivary profile also paralleled that observed in serum. There was a distinct difference in salivary cortisol concentrations during therapy in comparison to levels before and after. Following the completion of therapy, adrenal function recovered and returned to pre-treatment level, i.e. subclinical hypoadrenalism where the second afternoon cortisol peak was lost (additional file 1, Figure 1). In this case, salivary cortisol levels appear to be an additionally useful tool in the assessment of HPA axis. In ASTs, it compares satisfactorily with serum cortisol in detecting AI. Although AI is best assessed by serum cortisol dynamics, this report strongly suggests that salivary concentration dynamics are a reliable and dependable tool in the diagnosis of AI. It is additionally convenient for patients, especially in the ambulatory setting. This assay is not widely and routinely available however. The loss of diurnal variation in salivary concentration also remains to be validated but is also indicative of AI. Our testing frequency may not have detected the second diurnal peak which may occur anywhere between 15:00 and 18:00 hours. Frequent samplings may be more revealing but at the risk of non-compliance. The absolute morning salivary cortisol concentrations of less than 3.8 nmol/L is a good indicator of (impending) adrenal failure [7,8]. This level declined steadily following IFN therapy indicating a continuing decrease of adrenal reserve where it is maximally stimulated by ACTH as indicated by the ACTH failure to rise further with progressive hypoadrenalism.

**Figure 1:**
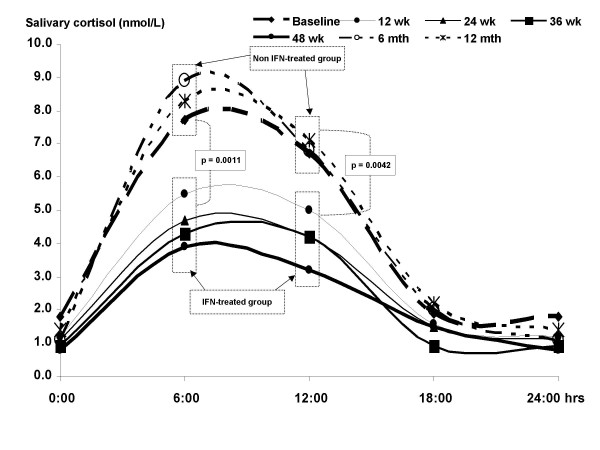
**Twenty-four hour salivary cortisol profiles *before, during and after *the patient underwent combination Interferon-α2β and Ribavirin therapy. **There is a significant difference between the IFN-treated vs. non-IFN treated groups.

The ACA titres peaked at the 48^th ^week. Thereafter, it returned to pre-treatment titre, paralleling the cortisol profiles.

The pathogenesis of HCV associated adrenal disease remains unknown and the molecular basis by which this occurs is undelineated. Normal CT imaging excluded anatomical causes such as haemorrhage, disseminated carcinoma or rarely antiphospholipid syndrome [9]. In autoimmune adrenal disease, it is thought that there must be an antigen presented on the adrenal cortical cells to stimulate the immune response. This response includes both T and B cells although the major determinant is in the former. The immune system is often stimulated by HCV infection, further amplified by IFN therapy. This is reflected by the positive but low titre of ACA at baseline, peaking at the nadir of adrenal function. This observation is consistent with a previous report [10] although our case additionally details the effect of the rise in ACA titre on cortisol metabolism. It is possible that IFN directly inhibits the synthetic function of adrenal cells and the swinging cortisol dynamics before and during treatment represents a balance between the inhibiting effect of IFN and the trophic effect of ACTH. Alternatively, the IFN may act by stimulating the T cells to destroy more adrenal cortical cells. ACTH was almost at its peak level pre-treatment and consequently failed to rise adequately to counter the adrenalytic effect of IFN. Other case report suggested an increase in cortisol consumption in AI proper [11]. Furthermore, the natural history of adrenalitis is unknown including its reversibility, the influence of IFN therapy and response to glucocorticoid supplement. Reversible autoimmune adrenalitis treated with high dose glucocorticoid associated with Graves' disease was previously described but this was distinctly different from our case [12]. The ACA titres parallel the dynamic of cortisol levels, peaking at the nadir of adrenal function although the absolute titre is modest. Nevertheless, this observation lends strong evidence to the immunopathogenesis of this condition.

Although the entity of subclinical AI has been well described [13], its management remains controversial. In *de novo *autoimmune situation, its progress depends on a number of factors such as ACA titres, the degree of adrenal gland failure, age and gender of the patient [14,15]. In the presence of hepatitis C infection, the progress is unknown although it can be surmised that the risk is higher due to the presence of ACA antibodies and subsequent interferon therapy. It is debatable as to the true 'subclinical' state of the condition given the presence of vitiligo. The patient however, had no other adrenal related symptoms although admittedly in the presence of HCV and IFN therapy, the clinical symptoms of adrenal failure can be very hard to elicit.

It was both tempting to treat this patient with replacement hydrocortisone given the biochemical results and the difficulties in separating adrenal failure symptoms and treatment related side effects. The latter made it very difficult to determine the active component of the symptomatology due to adrenal insufficiency. Treatment with supraphysiological corticosteroid has been reported to reverse the condition in a similar clinical scenario but in the absence of HCV infection [12]. Physiological replacement, which was non-immunosuppresive, was not required as the patient has adequate baseline cortisol, but emergency supraphysiological supplement was made available in the contingency plan.

## Conclusion

This case explores the immunopathogenic effect of hepatitis C virus and IFN therapy on the adrenal cortex. It is made more interesting by the presence of incidental and subclinical AI. Whilst clinically silent, the clinical scenario allows for the exploration of the use of salivary cortisol levels which appears to be a useful tool in detecting AI, either in a AST or a 24-hour profile.

## Competing interests

The authors declare that they have no competing interests.

## Authors' contributions

HAT provided the clinical case, consented the patient, conceived the study, participated in its design, assisted with data collection and statistical analysis, and coordinated and helped to draft the manuscript. SS and RJL analysed, provided the data, and participated in the discussion and drafting of the manuscript. GEMR contributed to the statistical and statistical analysis, participated in the discussion and drafting of the manuscript. All authors read and approved the final revised manuscript.

## Consent

Written informed consent was obtained from the patient for publication of this case report and biochemical data. A copy of the written consent is available for review by the Editor-in-Chief of this journal.

## Supplementary Material

Additional file 1 Table 1. Salivary and plasma cortisol, ACTH and ACA levels at various time points of IFN-based therapy Click here for file
