# Supplementary material for: Exacerbation of hepatitis C induced subclinical hypoadrenalism by Interferon-alpha2beta: A case report
Source: Cases J. 2008 Sep 18;1:157. doi: 10.1186/1757-1626-1-157 (PMC2556317; doi:10.1186/1757-1626-1-157)
Supplement: Additional file 1 — Table 1. Salivary and plasma cortisol, ACTH and ACA levels at various time points of IFN-based therapy [file 1757-1626-1-157-S1.doc]

Table 1.

|  | Salivary Cortisol following Synacthen testing (nmol/L) | | | Plasma Cortisol following Synacthen testing (nmol/L) | | | ACTH  (RR, <10.4 pmol/L) | 24 hour salivary cortisol profile  (nmol/L) | | | | Adrenal Cell auto-antibodies |
| --- | --- | --- | --- | --- | --- | --- | --- | --- | --- | --- | --- | --- |
| Time | 0 | +60 | **Absolute increment**  **(nmol/L)** | 0 | +60 | Absolute increment  **(nmol/L)** |  | 00:00 | 06:00 | 12:00 | 18:00 |  |
| At diagnosis | 5.2 | 6.1 | 0.9 | 481 | 529 | 48 | 67.3 | 1.8 | 7.7 | 6.7 | 1.9 | 1:40 |
| Twelve weeks | 4.4 | 4.6 | 0.2 | 419 | 433 | 14 | 87.9 | 1.2 | 5.5 | 5.0 | 1.6 | 1:40 |
| Twenty four weeks | 4.5 | 3.8 | ***0.0*** | 402 | 397 | ***0*** | 82.6 | 1.1 | 4.7 | 4.2 | 1.5 | 1:160 |
| Thirty six weeks | 3.3 | 3.8 | 0.5 | 342 | 356 | 12 | 72.6 | 0.9 | 4.3 | 4.2 | 0.9 | 1:160 |
| Forty eight weeks (completion of treatment) | 3.1 | 3.7 | 0.6 | 367 | 358 | ***0*** | 82.8 | 0.8 | 3.9 | 3.9 | 1.5 | 1:640 |
| At 6-month follow-up | 5.3 | 6.4 | 1.1 | 479 | 523 | 44 | 52.5 | 1.1 | 8.9 | 6.8 | 2.0 | 1:80 |
| At 12-month follow-up | 6.0 | 6.3 | 0.3 | 495 | 539 | 44 | 61.3 | 1.4 | 8.3 | 7.1 | 2.2 | 1:80 |
| *Mean* cortisol concentration *before and after* IFN treatment | 5.5 | 6.3 |  | 485.0 | 530.3 |  |  |  | 6.2 | 5.2 |  |  |
| *Mean* cortisol concentration *during* IFN treatment | 3.8 | 4.0 |  | 382.5 | 386.0 |  |  |  | 4.6 | 4.2 |  |  |
| *P values ** | < 0.01 | < 0.01 |  | < 0.01 | < 0.01 |  |  |  | < 0.01 | < 0.01 |  |  |

Salivary and plasma cortisol, ACTH and ACA levels at various time points of IFN-based therapy. Where increment is *negative*, zero change is inserted. *, P values were derived using the Student’s t-test, the means were for unpaired samples with unequal variance, (α = 0.05).
